# Enhertu as an effective treatment for metastatic lung cancer with brain metastasis recurrence: a case report

**DOI:** 10.1097/MS9.0000000000003921

**Published:** 2025-09-17

**Authors:** Wania Mohammad Akram, Swetha Kannan, SH Tirmazy, Dina Hamza

**Affiliations:** a Thumbay University Hospital, UAE; b Department of Oncology, Dubai Health, UAE; c Oncology, Mohammed Bin Rashid University of Medicine and Health Sciences (MBRU), UAE

**Keywords:** adenocarcinoma, brain metastasis, case report, clinical response, Enhertu, HER2-positive, metastatic lung cancer, nonsmall cell lung cancer (NSCLC), recurrent metastasis, targeted therapy, trastuzumab deruxtecan

## Abstract

**Background:**

Metastatic lung cancer with recurrent brain metastasis presents significant treatment challenges. Enhertu (trastuzumab deruxtecan), a targeted therapy for HER2-positive cancers, has shown promise in treating solid tumors, including nonsmall cell lung cancer (NSCLC) with brain metastases. This case report examines the efficacy of Enhertu in a patient with metastatic lung cancer and recurrent brain metastasis.

**Case presentation:**

A 39-year-old woman diagnosed with Stage IV adenocarcinoma of the left lung, negative for EGFR, ALK, and ROS-1, but with an ERBB2 mutation, experienced neurological symptoms leading to a diagnosis of brain metastasis in 2019. After multiple lines of therapy, including surgery, irradiation, chemotherapy, and targeted treatments, she presented with recurrent brain metastasis in January 2024. Following progression despite previous treatments, she was started on Enhertu in February 2024.

**Intervention:**

The patient was treated with Enhertu, and after six cycles, radiologic evaluation via PET and MRI scans in June 2024 demonstrated significant reduction in the size of cerebral and cerebellar metastases, with no new lesions or abnormal meningeal enhancement. Additionally, there was notable regression in the size and metabolic activity of lung lesions and previously hypermetabolic nodes.

**Results:**

Enhertu led to a partial response, with regression of most metastatic sites, including brain and lung metastases, and no evidence of new hypermetabolic lesions. The treatment was well tolerated with minimal adverse effects.

**Conclusion:**

Enhertu represents a promising treatment option for HER2-mutant metastatic lung cancer with recurrent brain metastasis. This case highlights the potential of Enhertu in overcoming treatment resistance in complex metastatic settings, warranting further investigation in broader clinical settings.

## Background

Lung cancer is one of the most common and deadly cancers worldwide, with a high incidence, particularly among smokers. The primary causes include tobacco smoke, environmental pollutants, and genetic factors. Early diagnosis can be challenging, as symptoms often appear in later stages; common diagnostic methods include chest X-rays, CT scans, and biopsies. Treatment options depend on the stage and type of lung cancer and may include surgery, radiation, chemotherapy, targeted therapy, and immunotherapy. Despite advancements, the prognosis remains poor, particularly for advanced-stage lung cancer, highlighting the importance of prevention and early detection^[1–3]^.

**HIGHLIGHTS**

- Enhertu (trastuzumab deruxtecan) induced a strong partial response in a patient with HER2-mutant metastatic lung adenocarcinoma.
- The patient demonstrated significant regression of recurrent brain metastases after multiple prior treatment failures.
- Enhertu was well tolerated with minimal side effects, offering a viable salvage therapy option.
- This case supports the expanding role of HER2-targeted therapies beyond breast and gastric cancers.
- Enhertu may be effective in overcoming resistance in heavily pretreated NSCLC with ERBB2 mutations.

Brain metastasis from lung cancer occurs when cancer cells from the lungs spread to the brain, a common complication in advanced stages of the disease. It is more prevalent in nonsmall cell lung cancer (NSCLC) and small cell lung cancer. Symptoms can include headaches, seizures, weakness, cognitive changes, and sensory disturbances. Diagnosis typically involves imaging techniques such as MRI or CT scans, along with biopsies if needed. Treatment options may include surgery, radiation therapy (such as whole-brain or stereotactic radiotherapy), and systemic therapies like chemotherapy, targeted therapy, or immunotherapy, depending on the specific characteristics of the cancer. Despite treatments, brain metastasis often indicates an advanced stage of lung cancer, making prognosis less favorable^[2,4]^.

Enhertu (trastuzumab deruxtecan) is a targeted therapy combining a monoclonal antibody and a chemotherapy drug, designed for HER2-positive cancers. Recently, it has shown promising results in treating metastatic cancers, particularly in cases with brain metastases, which are challenging to manage. In lung cancer patients, especially those with HER2 mutations, brain metastasis often represents a significant hurdle in treatment and prognosis. This case report explores the efficacy of Enhertu in a patient with metastatic lung cancer and recurrent brain metastases, offering insight into its potential as a treatment option for such complicated presentations. Through a detailed analysis, we aim to evaluate the clinical outcomes and therapeutic benefits of Enhertu in this unique setting^[5,6]^.

## Case report

A 39-year-old woman was diagnosed with Stage IV adenocarcinoma of the left lung, which tested negative for EGFR, ALK, and ROS-1 mutations, while showing a 2% expression of PD-L1 and an ERBB2 mutation. Her medical journey began in April 2019 when she experienced neurological symptoms that led to the discovery of a left frontal lobe mass. A craniotomy performed in May 2019 identified the mass as adenocarcinoma, potentially originating from the lung. This diagnosis was confirmed with a subsequent craniotomy in June 2019. The patient underwent a left upper lobe trisegmentectomy in July 2019, followed by whole-brain radiation in August 2019. In September 2019, she began four cycles of cisplatin and navelbine. Disease recurrence in August 2021 led to treatment with pembrolizumab, carboplatin, and pemetrexed, which continued until December 2021. Maintenance therapy with pembrolizumab and alimta followed until May 2022. From May 2022 to March 2023, she received ramucirumab and docetaxel, but developed ototoxicity after a cycle of carboplatin and gemcitabine in March 2023. She then switched to gemcitabine and herceptin in April 2023. PET scans from July and November 2023 showed mixed responses, with some lesions stable and others still active. In December 2023, she experienced new symptoms, and an MRI in January 2024 revealed recurrent brain metastases. Palliative radiotherapy for the neck and left supraclavicular lesion was recommended, with further treatment to be based on future PET scan results^[7–9]^.

She underwent palliative radiotherapy to the neck but did not receive brain radiation due to reaching the maximum dose. In February 2024, she began treatment with Enhertu. After six cycles of Enhertu, a PET scan and MRI in June 2024 showed significant shrinkage of multiple cerebral and cerebellar metastatic deposits, with no new meningeal enhancement and stable background lesions. Postoperative changes included left frontal encephalomalacia, diffuse cerebral atrophy, and unchanged periventricular high T2/FLAIR signal intensity. The updated PET scan revealed notable regression in the size and metabolic activity of hypermetabolic nodes in the left supraclavicular, mediastinal, and hilar regions, as well as lung nodules. There were no new FDG-avid lesions, and most metastatic bone lesions showed metabolic resolution, with mild activity remaining in the right iliac bone and left femur shaft. There was heterogeneous FDG uptake in the liver and pelvic areas, with reduced focal FDG avidity in the right uterine adnexa. Overall, a good partial response was observed, with regression of most metastatic sites and no evidence of new hypermetabolic lesions^[9–11]^.

The following images demonstrate her PET-CT scans before and after Enhertu treatment.

**Figure 1.**
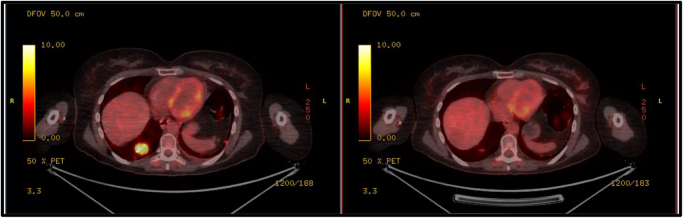
Baseline axial FDG-PET/CT image demonstrating intense hypermetabolic activity within mediastinal and hilar lymph nodes, consistent with metabolically active disease prior to initiation of trastuzumab deruxtecan (Enhertu) therapy.

Figure 1 is the baseline axial FDG-PET/CT image demonstrating intense hypermetabolic activity within mediastinal and hilar lymph nodes, consistent with metabolically active disease prior to initiation of trastuzumab deruxtecan (Enhertu) therapy.

**Figure 2.**
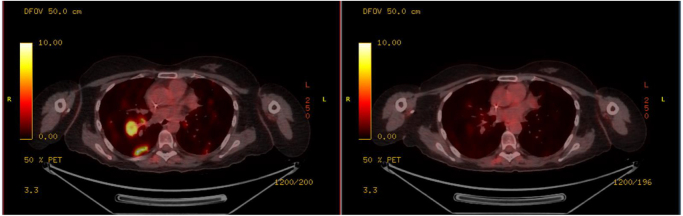
Interval axial FDG-PET/CT following treatment with trastuzumab deruxtecan showing marked reduction in FDG uptake, indicative of a partial metabolic response.

Figure 2 shows the interval axial FDG-PET/CT following treatment with trastuzumab deruxtecan showing marked reduction in FDG uptake, indicative of a partial metabolic response.

**Figure 3.**
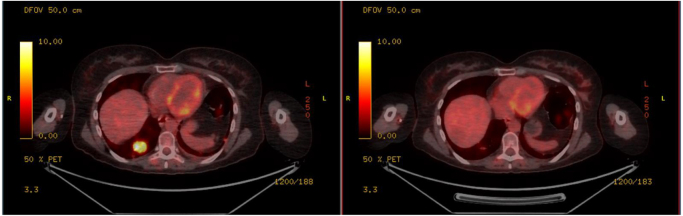
Comparative axial PET–CT slices before and after trastuzumab deruxtecan therapy illustrating significant decrease in metabolic activity of previously involved nodal regions.

Figure 3 highlights the comparative axial PET-CT slices before and after trastuzumab deruxtecan therapy illustrating significant decrease in metabolic activity of previously involved nodal regions.

## Discussion

Enhertu is an antibody-drug conjugate (ADC) designed to treat cancers that overexpress the HER2 protein. It works by binding to the HER2 receptor on cancer cells, delivering the chemotherapy drug directly to the tumor, which helps to minimize damage to healthy cells while killing cancer cells. Enhertu is primarily used to treat HER2-positive breast cancer and gastric cancer, but it has shown promising results in treating other HER2-positive tumors, including NSCLC and other solid tumors^[6,12]^.

Common side effects include nausea, fatigue, hair loss, diarrhea, and low blood cell counts. More serious adverse events can include interstitial lung disease (ILD) and cardiotoxicity. Given its ability to target HER2-positive cancer cells effectively, Enhertu has emerged as an important option in the treatment of advanced or metastatic cancers, offering hope for patients with limited treatment options.

The first tumor-agonistic approval of a HER2-directed therapy and ADC by FDA was based on the results from the subgroup of patients with HER2-positive Immunohistochemistry 3 + tumors in Destiny-Pantumor02, Destiny-Lung01, and Destiny-CRC01 phase II trials.

## DESTINY-Breast01 trial (HER2-positive metastatic breast cancer)[12]

The DESTINY-Breast01 study was a phase 2, multicenter, open-label study designed to evaluate the efficacy and safety of trastuzumab deruxtecan in patients with HER2-positive metastatic breast cancer who had progressed after multiple prior therapies, including trastuzumab, taxanes, and capecitabine. In the study, Enhertu demonstrated an overall response rate (ORR) of 60.9% in patients previously treated with other HER2-targeted therapies. The median progression-free survival (PFS) was 16.4 months, and overall survival (OS) data were also encouraging (Modi et al., 2020). Enhertu has demonstrated high efficacy in heavily previously treated patients, with many patients achieving durable responses. However, the study also highlighted the risk of ILD, a known adverse effect of trastuzumab deruxtecan, which led to some patients discontinuing treatment due to pulmonary toxicity.

## DESTINY-Breast03 trial (HER2-Positive metastatic breast cancer)[13]

The DESTINY-Breast03 trial was a phase 3, randomized, open-label study comparing trastuzumab deruxtecan with T-DM1 (another HER2-targeted ADC) in patients with HER2-positive metastatic breast cancer who had previously received trastuzumab and a taxane. The trial demonstrated that trastuzumab deruxtecan was significantly more effective than T-DM1 in terms of PFS. The median PFS for patients treated with Enhertu was 16.4 months compared to 6.8 months for patients treated with T-DM1. The ORR for Enhertu was 79.7% compared to 34.2% for T-DM1. These results strengthen Enhertu’s position as a preferred option for patients with HER2-positive metastatic breast cancer. However, the study also revealed safety concerns, particularly the risk of ILD, which occurred in some patients treated with Enhertu and could be managed with close monitoring.

## DESTINY-PanTumor02 trial[14]

The DESTINY-PanTumor02 trial is a pivotal phase II study evaluating the efficacy and safety of trastuzumab deruxtecan (T-DXd), an antibody-drug conjugate targeting HER2, in patients with advanced HER2-expressing solid tumors, including endometrial, cervical, ovarian, bladder, biliary tract, pancreatic, and other cancers. The study found that T-DXd showed significant objective response rates (ORR) of 51.4% in Immunohistochemistry 3 + tumors and 26.5% in Immunohistochemistry 2 + tumors. Duration of response (DOR) was also notable, with Immunohistochemistry 3 + tumors showing a median DOR of 14.2 months and Immunohistochemistry 2 + tumors showing 9.8 months. The safety profile was consistent with previous findings for T-DXd, with a noted risk of ILD. These findings demonstrate that T-DXd provides durable clinical benefits and meaningful survival outcomes, particularly in Immunohistochemistry 3 + tumors, supporting its role as a tumor-agnostic therapy for patients with HER2-expressing solid tumors and offering an effective treatment option for patients with limited alternatives.

## DESTINY-Breast04 trial (HER2-Low breast cancer)[15]

The study of Destiny-BREAST04 was a third phase study, and was evaluated by the effectiveness of the bullstakan trasumab in HER2-MOD breast cancer patients [Immunohistochemistry] 1 + or with a negative fluorescent hybridization. Hybridization is defined as 2 + [fish]). This study has expanded the potential group of patients for information that was previously limited to positive HER2 cancer. In this study, trastuzumab deruxtecan demonstrated significant improvements in progression-free survival and overall survival compared to chemotherapy in the HER2-low cohort. Median PFS was 9.9 months for patients treated with Enhertu, compared with 5.1 months for those treated with chemotherapy. The objective response rate (ORR) in the Enhertu group was 37.1%, indicating a significant benefit for patients with low HER2 gene expression. These results suggest that trastuzumab deruxtecan may be an important treatment option for a broader population of breast cancer patients who have not previously had targeted therapy options.

## DESTINY-Gastric 01 trial (HER2-Positive gastric cancer)[16]

The DESTINY-Gastric01 study evaluated the efficacy and safety of trastuzumab deruxtecan in patients with HER2-positive gastric or gastroesophageal junction cancer who had received at least two prior lines of therapy. This Phase 2 study demonstrated superior efficacy in a patient population with limited treatment options. The study results showed an ORR of 51.3% and a median overall survival of 12.5 months (Shitara et al., 2020). This is revolutionary, as patients with advanced gastric cancer generally have a poor prognosis and limited treatment options. Trastuzumab deruxtecan provided significant clinical benefit and improved survival outcomes in this population. Despite its efficacy, the risk of ILD remains a concern and requires careful monitoring during treatment.

## HER2-Positive colorectal cancer[17]

Colorectal cancer (CRC), especially positive CRC HER2, is an area where Erasel has the potential. HER2 amplification is less frequent in CRC than breast cancer, but is discovered in the patient’s subset, and these patients have in principle poor prognosis. The DESTINY-CRC01 trial tested trastuzumab deruxtecan in patients with HER2-positive metastatic CRC who had received at least two prior chemotherapy regimens. The study showed that trastuzumab deruxtecan produced an ORR of 32.2% in this cohort (Modi et al., 2021). Notably, the trial also showed promising PFS, with a median PFS of 5.6 months. These results were particularly important because they offered a new treatment approach for HER2-positive CRC, a cancer type that typically does not respond well to traditional HER2-targeted therapies. However, as in other studies, ILD remained a concern in this study.

## HER2-Positive endometrial cancer[18]

Besides gastrointestinal and breast cancers, HER2-positive endometrial cancer is also a potential indication for Enhertu, which can show HER2 overexpression, especially in more aggressive subtypes. Although this type of cancer is less frequently associated with HER2 positivity, a significant proportion of patients with progressive or recurrent disease may benefit from HER2-targeted therapy. A phase 2 study (DESTINY-Endometrial) evaluated the efficacy of trastuzumab deruxtecan in patients with HER2-positive, recurrent or metastatic endometrial cancer. The first data suggest that Uphetu can provide considerable clinical benefits. In this test, a 25–30% ORR was reported in patients with positive HER2 tumors. The results of this study suggest that ENHARTZ may be a treatment option for patients with advanced HER2-positive endometrial cancer, especially those with a poor prognosis.

## HER2-Mutant NSCLC[19]

One of the exciting developments in the clinical application of Enhertu is its activity in HER2-mutant NSCLC. Although HER2 mutations are not as common as other mutations like EGFR or KRAS in lung cancer, the discovery of effective therapies targeting HER2 mutations has been a breakthrough in lung cancer treatment. The DESTINY-Lung02 trial, which builds on the findings of the DESTINY-Lung01 trial, explored trastuzumab deruxtecan’s efficacy in HER2-mutant NSCLC patients. The DESTINY-Lung01 study demonstrated an overall response rate (ORR) of 55.4%, and the findings from DESTINY-Lung02 further corroborated these positive results, with similar ORRs reported for patients with HER2 mutations in NSCLC. The clinical outcomes in these patients were particularly significant given the poor prognosis and limited treatment options available to this population.

### Other studies

In a small pilot study published in *Gynecologic Oncology*, trastuzumab deruxtecan was tested in women with HER2-positive ovarian cancer who had previously received chemotherapy. The results were promising, with some patients showing partial responses and a manageable safety profile, though ILD was again observed as a potential risk (Briggs et al., 2021). These early findings suggest that trastuzumab deruxtecan could be a useful treatment for HER2-positive ovarian cancer, although larger studies are needed to confirm these results.

One such trial is the DESTINY-Breast07 20 trial, which is investigating the combination of trastuzumab deruxtecan with immune checkpoint inhibitors like pembrolizumab in HER2-positive breast cancer patients. Early data suggest that this combination could improve overall survival and reduce the risk of progression, although safety concerns, particularly regarding ILD, are closely monitored (Bianchini et al., 2022). This combination approach is being considered in several solid tumor types, including **gastric** and **lung cancers**, where HER2-targeted therapies are combined with immunotherapy to exploit potential synergistic effects.

## Conclusion

In conclusion, this case report highlights the promising potential of **Enhertu** as a treatment for lung cancer patients with ERBB2 mutations and HER2 overexpression, particularly in the context of recurrent brain metastasis. The patient’s positive response to Enhertu, marked by significant regression of both brain and lung lesions, demonstrates the efficacy of this targeted therapy in overcoming resistance to previous treatments. This case provides valuable insight into the utility of Enhertu for lung cancer patients with specific genetic mutations and brain metastases, advocating for its consideration in future clinical strategies for this challenging patient population.

## Data Availability

No datasets were generated.

## Research registration unique identifying number (UIN

This is not a research. It is a case report.
